# Smoking as a persistent global health challenge: perspectives on heated tobacco products

**DOI:** 10.3389/fpubh.2026.1737290

**Published:** 2026-09-21

**Authors:** Anadi Bisht, Shuki, Manish Singh, Ritu Kulshreshtha

**Affiliations:** 1 School of Interdisciplinary Research, Indian Institute of Technology Delhi, New Delhi, India; 2 Department of Biochemical Engineering and Biotechnology, Indian Institute of Technology Delhi, New Delhi, India; 3 School of Public Policy, Indian Institute of Technology Delhi, New Delhi, India; 4 Center for Environmental Assessment and Climate Change, G.B. Pant National Institute of Himalayan Environment, Kosi-Katarmal, Almora, Uttarakhand, India

**Keywords:** biomarkers of potential harm, e-cigarette, harm reduction, health burden, heated tobacco, smoking, tobacco control policy, tobacco products

## Abstract

Smoking remains one of the leading preventable causes of morbidity and mortality worldwide, significantly contributing to the healthcare burden. Despite decades of public awareness, many people are unable to quit combustible cigarette (CC) smoking due to nicotine dependence and/or limited access to effective cessation support. Various alternative tobacco products such as e-cigarettes, heated tobacco products (HTPs) and nicotine pouches have been promoted as lesser harm alternatives; among which HTPs have received increasing attention lately. However, these products differ substantially in their composition, patterns of use, and potential health effects. This narrative review evaluates evidence on HTPs relative to CCs across four distinct levels: (i) product emissions, (ii) biomarkers of human exposure, (iii) indicators and evidence relevant to individual disease risk, and (iv) potential population-health impact. These levels are considered separately because reduced emissions or exposure cannot, by itself, establish reduced clinical disease, mortality, or net population benefit. Compared to combustible cigarettes, HTPs may lessen exposure to some combustion-related toxicant, but they are not risk-free; their population-health impact depends on who takes up these products and under what social and cultural circumstances, the frequency, intensity and duration of use, exclusive versus dual or poly use, users’ prior exposure and health status, and the wider institutional, infrastructural and regulatory environment. To guide effective policy formulation for harm reduction based on evidence further longitudinal epidemiological and policy-evaluation studies across diverse settings are needed to assess long-term health outcomes and determine how tobacco-control and harm-reduction strategies—including public communication and education, taxation, product regulation and cessation support—shape awareness, risk perceptions, product uptake and substitution, patterns and intensity of use, cessation and relapse.

## 1 Introduction

Worldwide, tobacco smoking led to more than 200 million deaths in the last three decades (1990–2019), placing severe burden on the global healthcare systems and the economy (1). The global economic cost of smoking-attributable diseases has been estimated at US$1.436 trillion, equivalent to 1.8% of global gross domestic product (2). Over 80% of the global 1.3 billion tobacco users live in low- and middle-income countries, where tobacco-related illness and death rates are the highest (3). Even passive smoking is associated with a high risk of coronary heart disease and cancer (4). Despite enforcing public health interventions, these countries continue to have disproportionate burden of high tobacco consumption; hence interventions must be tailored after considering the regional and national disparities (5). The persistent and unevenly distributed burden of smoking underscores the urgent need to strengthen and equitably implement comprehensive, evidence-based tobacco-control measures (1).

Alternative tobacco products such as electronic cigarette (EC), smokeless oral products, snus and heated tobacco products (HTPs) for being potentially less harmful, are offered to diminish the growing health burden of traditional combustible cigarette (CC) smoking (6). Among them, HTPs become increasingly dominant in many markets due to their claim of delivering nicotine without combustion and reducing exposure to harmful combustion by-products (7). HTPs are just one of a wide range of nicotine products available, including disposable nicotine/ nicotine-free vapes, refillable electronic cigarettes (including nicotine-salt formulations), nicotine pouches, snus and myriad oral smokeless products, and prescription nicotine replacement therapies (NRTs). The nicotine pharmacokinetics, abuse liability, juvenile appeal, toxicity, and potential for dual or polysubstance use (e.g., contemporaneous THC and nicotine vaping or combined oral nicotine + cannabis products) of these items vary significantly. Evaluating HTPs alone runs the risk of oversimplifying current nicotine exposure patterns and public health implications because many users, especially teenagers and young adults, may embrace many products or switch between them. The Food and Drug Administration, in April 2019, has authorized the sale of HTP and in 2020, permitted its marketing as an alternative reduced-risk tobacco product to CC (8). However, the extent to which HTPs reduce health risks compared to smoked tobacco consumption and whether the HTPs are truly less harmful remain a subject of discussion (9). The central question of this narrative review is: What does the current evidence indicate about the effects of HTPs compared with continued CC smoking, particularly with respect to emissions, human exposure, biomarkers and other indicators of disease risk, and potential population-health outcomes? What key evidentiary gaps limit the strength of current conclusions? Cessation effectiveness is considered as a separate outcome and is not inferred from reduced emissions or exposure. Reduced emissions refers to lower measured yields of selected constituents under specified testing conditions; reduced exposure refers to lower internal dose measured through biomarkers; reduced individual disease risk requires evidence linking product use to clinical outcomes over adequate follow-up; and reduced population harm requires integration of product-specific risk with uptake, complete switching, dual or poly use, cessation, relapse, and initiation. Evidence at one level is not to be interpreted as proof at a higher level.

### 1.1 Harm reduction as opposed to harm elimination

Differentiating between damage reduction and harm removal is crucial. The goal of harm reduction is to lessen the health risks associated with nicotine use while acknowledging that some people will continue to use it (e.g., by substituting non-combustible items that produce less combustion toxicants). The goal of harm elimination is for everyone to completely stop using tobacco and nicotine. Although HTPs do not eliminate harm, they may be useful as a harm-reduction strategy for adult smokers who would otherwise continue to use combustible cigarettes. Therefore, policy choices should weigh the hazards of beginning, long-term nicotine dependency, and uptake among adolescents and non-smokers against the possible benefits for adult smokers who quit entirely.

## 2 Methodology

This work is a narrative review of the scientific literature on the global smoking epidemic and harm reduction strategies, with a focus on heated tobacco products (HTPs). A narrative approach was intentionally adopted to synthesise evidence across heterogeneous study designs, populations, and endpoints, including mechanistic, clinical, and policy-level perspectives, that would not be amenable to quantitative pooling. The narrative design was used to integrate heterogeneous evidence across four pre-defined evidentiary levels. The review does not estimate a pooled relative risk, establish comparative cessation efficacy, or model net population-health impact. Its conclusions are descriptive and calibrated to study design, comparator, endpoint, duration, and product-use pattern. Limitations inherent to this approach, including potential for selection bias and inability to formally assess publication bias, are acknowledged.

Literature searches were conducted across PubMed and Google Scholar. Searches were performed using the combination of keywords, explored independently to maximise retrieval of relevant literature: “smoking,” “tobacco products,” “global perspective,” “health burden,” “smoke emission,” “secondary exposure,” “biomarkers of potential harm,” “harm reduction strategy,” “e-cigarettes,” “heated tobacco,” and “policy and prevention.” Searches were performed using combinations of keywords connected with Boolean operators (AND/OR) to maximize retrieval; however, the search was not designed to meet systematic review reproducibility standards. The authors acknowledge that this approach may introduce incomplete coverage of the evidence base, which is a recognised limitation of narrative reviews.

Articles were included if they met all of the following criteria: (i) full-text available in the English language; (ii) peer-reviewed studies published between 2020 and 2025; and (iii) focus on smoking-associated health burden, tobacco harm reduction approaches, smoke emissions, biomarkers of exposure or potential harm, e-cigarettes, heated tobacco products, or tobacco control policies. Articles were excluded on the following grounds: (i) non-English language publications; (ii) conference abstracts, editorials, letters, commentaries, and duplicate articles; and (iii) studies not directly relevant to the scope of the review.

Initial screening was performed by title and abstract review against the inclusion and exclusion criteria, followed by full-text assessment for eligibility. Data were extracted narratively by thematic category: smoke emissions, health risk and toxicology, molecular and cellular effects, biomarkers of exposure or potential harm, and regulatory and public health frameworks. A total of 65 articles met the selection criteria and were included in the review, comprising 4 systematic reviews and meta-analyses, 6 trials, 41 original research articles, 5 reports, and 9 review articles. A formal risk-of-bias assessment was not conducted given the narrative review design; however, the authors have noted relevant study-level limitations, including short follow-up durations, observational designs, and geographical concentration of evidence in Japan, the Republic of Korea, and selected European settings, where they bear on the interpretation of findings. A visual overview of the literature search and selection process is done by SANRA (Scale for the assessment of narrative review article) as presented in Figure 1.

**Figure 1:**
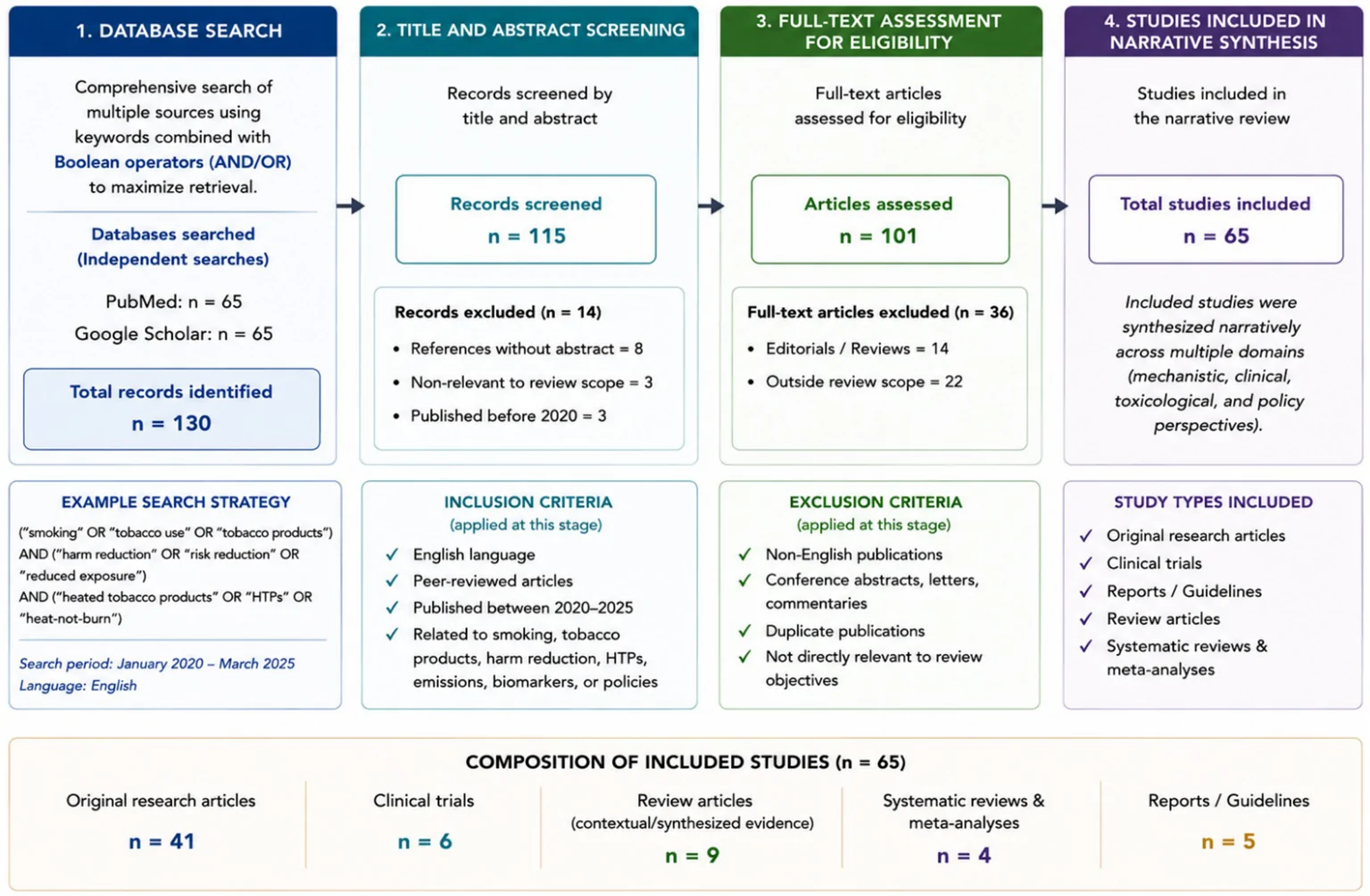
SANRA flow diagram illustrating the literature search and selection process for this narrative review.

Comparisons clearly specify the reference group: continued CC smoking, abstinence or never use, medicinal nicotine, or other non-combustible products. Evidence from one comparison is not applied to another, and dual or poly use is assessed separately from complete switching.

### 2.1 Overview of tobacco and nicotine products

#### 2.1.1 Traditional CCs

The basic components of the tobacco leaves such as biopolymers, carbohydrates, proteins and waxes, degrade at high temperature (650–900 °C) of cigarettes smoking, and release 6,000 compounds including 100 toxic compounds that are responsible for smoking-related diseases (10). Second-hand smoke (passive/involuntary), emitted from CCs or exhale from smokers that contain many carcinogens, is another major health concern for non-smokers, especially women and children (11). Each year, second-hand smoke kills ~1.6 million non-smokers (3). Additionally, the non-biodegradable cellulose acetate in cigarette filters if not discarded properly, contributes significantly to microplastic pollution, which affects the ecosystem (12). The harmful effects of both active and passive smoking on health and environment need to be addressed through public health policies and education.

#### 2.1.2 Alternative tobacco and nicotine products

The novel and emerging nicotine and tobacco products comprised EC [including electronic nicotine delivery system (ENDS) or electronic non-nicotine delivery systems], HTPs, nicotine pouches, smokeless oral products and snus have rapidly gained popularity among youths. Vapes or EC including ENDS use a battery to heat flavoured e-liquid solution. Nicotine pouches unlike tobacco-containing snus, carry nicotine powder, salts, sweeteners and flavourings (13). They are increasingly gaining popularity among young generation because of variety of flavours and targeted marketing strategies as reduced-risk alternatives (14). However, EC is related to depression even in absence of current or previous CC smoking (15), and can cause adverse respiratory symptom, burns, lung injury, poisoning and seizures (13). The HTPs are comparatively new, commonly called as heat-not-burn by the tobacco industry (16), contain various tobacco types and have diversity in heating style (13). Tobacco in HTPs is heated at a temperature of 300–350 °C to generate satisfactory amount of aerosols of nicotine, but without combustion, thereby reducing the formation of as well as exposure to the toxic substances (10). Popular HTP brands that are available in the market include IQOS by Philip Morris International, glo/iFuse by British American Tobacco and Ploom by Japan Tobacco International (6). The HTPs claim to yield ~30% lower nicotine than the CCs (17). Despite the claim, HTPs are banned in 11 countries including Brazil, Democratic People’s Republic of Korea, Ethiopia, India, Iran, Mexico, Norway, Panama, Singapore, Syrian Arab Republic and Timor-Leste); while in other countries, they are implicitly or explicitly regulated as tobacco products (18). Other alternatives are nicotine replacement therapy comprising nicotine patches, gum or lozeng that deliver controlled doses of nicotine to manage withdrawal symptoms during smoking cessation or smooth transition to complete abstinence (19). Alternative tobacco products are used by 0.5–5% of the adult population, 20% of which using them with intention to smoke reduction or cessation (20).

### 2.2 The environment of novel nicotine products and the use of polysubstance

The distribution of nicotine has quickly become more diverse. HTPs coexist in many markets alongside synthetic nicotine products, nicotine pouches (tobacco-free oral nicotine), disposable and refillable electronic nicotine delivery systems (ENDS), and nicotine salts that allow for nicotine administration with or without combustion. Exposure evaluation is made more difficult by the growing number of reports of polysubstance patterns. Furthermore, dual and poly use complicate the interpretation of biomarker and epidemiological findings, because measured exposure may arise from more than one product and may vary substantially according to the frequency and intensity of combustible-cigarette use. Also, since at the population level, widespread concurrent use may limit the overall public health benefits of tobacco harm reduction strategies and should therefore be considered a major confounding factor when assessing the risks and benefits of novel nicotine products. In a biomarker study comparing exclusive and concurrent product-use patterns, individuals concurrently using HTPs and combustible cigarettes had higher exposure to tobacco-related respiratory toxicants than exclusive HTP users, although exposure to some toxicants was moderately lower than among exclusive cigarette smokers (21). Studies of concurrent cigarette and e-cigarette use similarly demonstrate that toxicant exposure may remain comparable to, or in some cases exceed, that observed among exclusive cigarette smokers, with substantial heterogeneity across dual-use patterns (22, 23). Accordingly, exclusive, dual, and poly users should be analysed separately when estimating product-specific exposure and potential population-level harm reduction (24).

### 2.3 Smoke emission

Cigarette emission contains over 7,000 chemicals, of which, a minimum of 250 are harmful to humans. The smoke carries highly reactive free radicals (>10^16^ molecules per puff) in gas phase and long-lasting radicals in the particulate phase (25). One CC contains 17,667 μg total particulate matter, which is ~2-fold of the HTPs and contributes to tar content (26). The CC produces more gas-phase radicals (567.6 ± 78.3 pmol/puff), particulate phase radicals (73.9 ± 7.5 pmol/puff) and nicotine (189.5 ± 7.9 μg/puff) than any tobacco product (25). The CC smokers are exposed to high level of combustion components, the pyridine and 4-ethenylpyridine that are considered carcinogenic to humans (27). Cigarette smoke indoors is associated with lifetime cancer risk of >1 per 100,000; for HTPs, the risk is lower (28). The uranium in tobacco leaves generates two radionuclides polonium-210 and lead-210, after undergoing decay, which are responsible for tobacco-related diseases. Mainstream CC smoke has 7% lead-210 and more polonium-210 than the HTPs (13.6 ± 4.1% vs. 1.8 ± 0.3%) (29).

Incomplete combustion and thermal degradation of the compounds (glycerol, propylene glycol and triacetin) that are used to prevent drying of tobacco sheet of HTPs form different types of radicals. These are responsible for production of highly reactive genotoxic carbon/ nitrogen-centered radicals (30). More than 60% of HTP aerosol collected mass contains water, propylene glycol and glycerol, which contributes ~40% of the total particulate matter from CCs (17). The International Agency for Research on Cancer classified 2-furanmethanol and pyridine as possibly carcinogenic. In comparison to CC, higher levels of 2-furanmethanol along with furfural, 2(5H)-furanone and 5-methylfurfural are found in the mainstream smoke of HTPs (27). Davigo et al. (31) reported that HTP aerosol contains 55, 25%, 63–89% and 95–98% lesser nicotine, total particulate matter, carbonyl compounds (acetaldehyde, acetone, acrolein, formaldehyde, glyoxal and methylglyoxal) and tobacco-specific nitrosamines respectively, compared to CC. However, the level of toxicants varies between different HTP flavors. Concentrations of HTP smoke also play crucial role in cell growth (32). The HTP produces lower gas-phase radicals than the CC (~12 vs. 567.6 ± 78.3 pmol/puff); however, chronic exposure could damage critical cellular pathways and pose significant health risk (25).

Similar to CC, active or passive HTP exposure increases lipopolysaccharide and zonulin levels, thereby causing intestinal barrier dysfunction and systemic endotoxemia. This alteration is likely to increase cardiovascular risk, insulin resistance and metabolic syndrome (33). Secondhand tobacco exposure was estimated as 27.6% in children from a family of CC and HTP users, 22.9 and 3.1% in households using CC and HTP respectively, and 1.4% in households not using tobacco. Therefore, irrespective of the type, any tobacco product should be limited to protect children as well as other members from risks of second-hand smoking (34).

### 2.4 Health impacts of smoking

Chronic obstructive pulmonary disease, coronary heart disease, lung cancer, stroke and other chronic diseases are the common smoking-associated diseases that reduce 10-years life expectancy (20). Smokers are also at high risk of developing periodontal disease, inflammatory bowel disease, stomach ulcers, diabetes, gastrointestinal tract cancer and dementia. Moreover, second-hand smoke increases the risk of asthma, bronchitis, middle ear infections, pneumonia and other respiratory infections in children, and of miscarriage, fetal death and stillbirth in pregnant women (35). HTPs are not risk-free, and available studies report adverse cardiovascular and respiratory findings in the users (36, 74). However, the evidence summarized here is predominantly experimental, cross-sectional, observational, or based on surrogate endpoints; it does not establish the comparative incidence of cancer, cardiovascular disease, chronic obstructive pulmonary disease, or all-cause mortality among people who switch completely from CCs to HTPs. Moreover, the impact of CCs and HTPs on mental health remains a significant research gap. In addition, the relatively recent introduction of HTPs has limited the availability of long-term epidemiological studies, making it challenging to fully assess their chronic health effects and compare their long-term risks with those associated with conventional cigarette smoking. Addressing this gap through further research will provide valuable insights to strengthen tobacco control programs and public health policies.

#### 2.4.1 Health risk associated with long-term use

Cigarette smoking results in color changes of enamel with more pronounced change in the cementum and reduced Ca/P ratio of human maxillary premolars than the HTPs; however, both smokes have similar effects in changing surface morphology and reducing mineral content (37). HTPs also worsens periodontal health by affecting the probing depth and clinical attachment loss when encounter smoke and tobacco, compared to the non-smokers (38). A cross-sectional study reported that both CC and HTP smokers develop significant impairments in olfactory and gustatory functions, compared to the non-smokers. Lifetime exposure to smoking was negatively correlated to olfactory and gustatory function, along with sour, salty and bitter tastes (r = −0.317 to −0.585, *p* ≤ 0.002) (39). The HTP similar to CC, but at a more intensive exposure was reported to induce cytotoxicity, Nrf2-dependent oxidative stress and oxidative DNA damage in human epithelial pulmonary cells (40). The current literature is largely based on short-term experimental, cross-sectional, or observational studies. Consequently, the lack of long-term prospective epidemiological studies limits the ability to accurately determine the chronic disease burden and long-term safety profile of HTPs relative to CCs. Understanding and comparing the long-term effects of CCs and HTPs through well-designed longitudinal studies is therefore essential to assess their health risks and guide evidence-based public health policies. Studies have compared the odds of developing various diseases in HTP and CC smokers across different populations (Table 1). Overall, the studies summarized in Table 1 suggest that both CC and HTP use are associated with adverse respiratory and cardiovascular outcomes, including chronic obstructive pulmonary disease, hypertension, airway obstruction, respiratory symptoms, and allergic conditions. Although several studies indicate that HTPs may result in lower exposure to certain toxicants than CCs, the available evidence does not make HTPs risk-free. Importantly, most studies included in Table 1 are cross-sectional or observational in nature and originate predominantly from Asian populations. Consequently, residual confounding, including prior smoking history and dual-product use, may influence reported associations, and the generalizability of findings to other populations may be limited. There is a clinical-endpoint evidence gap. No long-term prospective evidence identified in this narrative review establishes that switching completely from CCs to HTPs reduces cancer, cardiovascular disease, chronic obstructive pulmonary disease, or mortality. Cross-sectional associations, short-term physiological changes, in-vitro findings, and biomarkers can inform biological plausibility and exposure assessment, but cannot establish long-term comparative clinical benefit.

**Table 1:** Health risks of using tobacco and nicotine-containing products.

Reference	Study type (country)	Sample size	Follow-up	Smoking history	Smoking history adjusted	Dual use adjusted	Disease risk	Funding
(63)	Cross-sectional (Italy)	22,428	NA	Dual and poly users of CC, EC and HTP	Yes	Yes	With CC smoking, dry cough was the most frequently reported adverse event, followed by sore throat, mouth dryness and vertigo	Non-industry [Italian Association for Cancer Research (AIRC), the Italian League Against Cancer (LILT), and the Joint Action on Tobacco Control 2 (JATC-2)]
							With EC and HTPs, mouth dryness followed by dry cough, sore throat were common events	
							CC smokers who used EC (AOR 1.32, 95% CI: 1.17–1.50) with HTP (AOR 1.14, 95% CI: 1.01–1.30) or both EC and HTP (AOR 1.50, 95% CI: 1.29–1.75) had higher chances of experiencing at least one adverse event, compared to only CC smokers	
(64)	Cross-sectional (Japan)	22,428	NA	Dual and poly users of CC, EC and HTP	Yes	Yes	57 recent events of COPD; smoking history affected the outcomes: OR 3.23 (95% CI: 1.37–7.61) without adjust for history vs. OR 1.85 (95% CI: 0.70–4.89) with adjustment	Industry-sponsored (Japan Tobacco Inc.)
							Asthma, cough, combined respiratory symptoms, lung function abnormalities, phlegm, shortness of breath and wheezing were the other health risks	
							It is important to adjust for previous smoking history while assessing health effect of HTPs	
(65)	Cohort (Japan)	30,152		12.3% CC, 9.4% HTP, 5.2% dual users	Yes	Yes	3,656 new cases of hypertension; higher among CC smokers (HR 1.26, 95% CI: 1.13–1.41) and HTP users (HR 1.19, 95% CI: 1.06–1.34) than non-smokers	Non-industry (Industrial Health Foundation, JSPS KAKENHI, NCGM Intramural Research Fund)
							Increased risk (HR 1.16, 95% CI: 0.98–1.38) of hypertension among dual users	
(66)	Single-center observational study (Japan)	2,850	NA	4.6% current HTP, 10.7% current CC users	Yes	No	Airway obstruction was rare among who stopped CC ≥ 5 years ago (APR 0.64, 95% CI: 0.46–0.89) than who stopped <1 year ago, and frequent among who currently used HTPs (APR 1.42, 95% CI: 1.002–2.00) than in who never used	Non-industry (Health Labour Sciences Research Grants; JSPS KAKENHI)
							Current HTP use led to airway obstruction (APR 2.32)	
(67)	KNHANES (2018–2020) data analysis (Republic of Korea)	18,230	NA	15% CC, 1% HTP, 2.4% dual users	Yes	Yes	Prevalence of COPD (AOR 2.37, 95% CI: 1.02–5.51) and allergic rhinitis (AOR 1.33, 95% CI: 1.13–1.57) was higher among past tobacco users than non-smokers	No external funding
							Prevalence of allergic rhinitis was higher among HTPs users than CC users (AOR 1.74, 95% CI: 1.14–2.66) or non-smokers (AOR 1.60; 95% CI: 1.06–2.42)	
							Prevalence of asthma was higher among past tobacco users (AOR 1.73, 95% CI: 1.26–2.38) and CC users (AOR 1.57, 95% CI: 1.08–2.26) than non-smokers	
(68)	Cross-sectional (China)	33,627	NA	Former/current CC, HTP, EC and other tobacco product users	Yes	Yes	16.3% had respiratory symptoms	Non-industry (Hong Kong SAR Government; Sir Robert Kotewall Professorship)
							Respiratory symptoms were more prevalent in ever HTP use (APR 1.46, 95% CI: 1.15–1.86) and ever dual use (APR 1.29, 95% CI: 1.08–1.54) than ever CC use	
							Respiratory symptoms were associated with former HTP use (APR 1.30, 95% CI: 1.06–1.59) and current HTP use (APR 1.59, 95% CI: 1.23–2.06) than who never used	
							No association between respiratory symptoms (APR 1.40, 95% CI: 0.93–2.11) and current HTP use	

AOR, Adjusted odds ratio; APR, Adjusted prevalence ratio; CC, Combustible cigarette; CI, Confidence interval; COPD, Chronic obstructive pulmonary disease; EC, Electronic cigarette; HTP, Heated tobacco product; HR, Hazard ratio; KNHANES, Korea National Health and Nutrition Examination Survey; OR, Odds ratio; NA, Not available.

#### 2.4.2 Toxicity, oxidative stress and inflammatory response

Smoke from both CC and HTP contains reactive oxygen species (ROS) and carbon/ nitrogen-centred radicals, but in different proportions. For instance, tobacco-specific nitrosamines that are higher in CC smoke than the HTPs, are mainly responsible for redox reactions and targets net Fpg-sensitive sites for DNA damage (30). Both HTP and CC smoke causes mitochondrial toxicity in respiratory epithelium by downregulating genes encoding oxidative phosphorylation, which in turn elevates ROS (41). A systematic review discussed that chronic exposure to CC smoke depletes the antioxidant defence (enzymatic/ non-enzymatic) during continuous action against oxidative burst. Chronic smoke either directly causes lung injury or induces various cellular responses by generating secondary metabolic reactive species (42). Cigarette smoke reduces human microglial viability, intracellular glutathione levels and expression of Klotho β (an aging marker), increases lipid peroxidation and ROS formation, and leads to DNA damage. In addition, it impairs mitochondrial function and unfolded protein response, and causes activation of nuclear factor-κB pathway, tumor necrosis factor and interleukin-18 expression, thereby inducing a pro-inflammatory state. On the other hand, the toxicity of HTP in microglia is comparatively less in terms of lipid peroxidation, ROS production and mitochondrial dysfunction (43). Cigarette smoke can elicit genotoxicity even at lower concentrations and promote DNA damage more than HTP emissions. However, the latter too has toxicological potential as it causes DNA strand breaks and alkali-labile site (30). Transcriptomic profile of bronchial and alveolar models exposed to HTP smoke demonstrated enriched oxidative stress pathways including α-tocopherol degradation, estrogen biosynthesis, ferroptosis, superoxide radical degradation, and xenobiotics metabolism signalling (44). Direct or second-hand CC or HTP exposure elevates NOX2 activation and hydrogen peroxide by increasing lipopolysaccharide level, and reduces nitric oxide bioavailability, p-selectin and sCD40L. This in turn amplifies the oxidative stress and platelet activation, and impairs the endothelial function (33). The HTP-associated complications like platelet clot formation, high inflammatory response, endothelial dysfunction and negative reproductive outcomes, are also noted in the mother (36). Pulmonary (bronchial and/or alveolar) toxicity by HTP smoke through lipid peroxidation, oxidative stress, DNA damage and ferroptosis was also reported (44).

#### 2.4.3 Molecular effects

Cigarette smoke elicits greater transcriptomic response via altering more than 1,000 gene expression, compared to HTPs (evoke <250 gene alterations) (41). The constant up-regulated genes across multiple tissues of CC smokers are xenobiotic metabolism-related *AHRR*, *CYP1A1* and *CYP1B1* (45). Traditional cigarette alters *MMP1*, *MMP2*, *MMP3* and *MMP9* expression as well as MMP3 protein levels, which weakens the plaque integrity and induces pro-inflammatory and pro-degradative environment (46). On the other hand, HTP and EC showed to improve anti-inflammatory response by upregulating *CHRNA7* gene, which encodes the α7 nicotinic acetylcholine receptor, a key mediator of the cholinergic anti-inflammatory pathway (46). Histone modification in CC smokers along with activation of apoptosis-related *EGR1*, *FOS* and *FOSB* genes are less likely to occur in vapor-generating HTP product users (47). However, it was also reported that CC, EC and HTP upregulate extracellular matrix-related *COL1A1*, *FN1*, *DCN* and *LUM* genes in human aortic smooth muscle cell, in turn promoting fibrosis and plaque progression, and reducing vessel dilation (46). Differential expression analysis showed altered expression of 72 circulating microRNAs in chronic traditional CC, 10 in HTP products, and 26 in both users, which are associated with many pathological conditions (48).

Smoking and aging have similar impact on gene expression and DNA methylation across human tissues (45). Transcriptomic profiling of young human aortic smooth muscle cells showed significantly higher biological impact of CC on smoking-dependent vascular aging and cardiovascular disorders than the HTPs (49). High concentration of HTP extract was found to increase the *CYP1A1* expression in lung epithelial cells that could be associated with high carcinogenic risk (32). A study showed increased *TET1* expression with HTP, but lesser influence on other DNA methylation-related gene expressions than the CC. Expression of lung cancer associated genes, *DKK1*, *GDF15*, *HMOX1*, *RASSF9* and *VEGFA* was altered by both HTP and CC (32). A cohort study reported hypomethylation of 10 out of 17 smoking-related DNA methylation biomarker genes (e.g., *AHRR*, *F2RL3*, *LRRN3*, *RARA* and *GPR15*) in CC and HTP users, compared to non-smokers. Compared with non-smokers, HTP users exhibited significantly lower DNA methylation levels (hypomethylation) of *LRRN3* and *GPR15*, while CC users showed particularly high expression of *AHRR* and *GPR15* (50). These findings suggest that tobacco-associated epigenetic alterations remain detectable in HTP users, reinforcing that smoking cessation is the most effective approach to improving long-term health and life expectancy (50).

### 2.5 Biomarkers of potential harm

Biomarkers of potential harm (BoPH) including F2-isoprostane, fibrinogen, forced expiratory volume in 1 s, high-sensitivity C-reactive protein, interleukin(IL)-6 and white blood cell count are typically used to assess health of smokers, effect of lower-risk tobacco products or of quitting smoking and improvement after quitting (36). Smokers who switch to HTPs or quit smoking showed to have improvement in levels of several BoPHs (36). Traditional cigarette increases expression of IL-1beta, IL-6 and IL-8, which are indicators of proatherogenic inflammatory profile; whereas, the expression of pro-inflammatory cytokine genes with EC and HTP use is lower (46). However, the HTPs also shown to elevate DNA damage markers, viz. 8-hydroxy-2′-deoxyguanosine, cleaved poly-(ADP-Ribose) polymerase and phosphorylated histone H2AX, in human bronchial and/or alveolar models (44). Cotinine, nicotine, trans-3′-hydroxycotinine and 4-(methylnitrosamino)-1-(3-pyridyl)-1-butanol in urine indicates exposure to second-hand smoke and HTP. Moreover, 7-methylguanine and 8-hydroxy-2′-deoxyguanosine are the DNA damage markers, level of which increase after second-hand exposure (51). Changes in biomarker levels following exposure to CC and alternative tobacco/ nicotine products are presented in Table 2.

**Table 2:** Biomarkers in traditional cigarette and alternative tobacco product users.

References	Sample size and smoking status	Biomarkers	Key findings	Clinical relevance
(54)	*n* = 224 (age: 20–65 years) regular CC adult smokers	TSNAs, VOCs and biomarkers for crotonaldehyde and acrolein were tested in urine sample (Biomarkers of Exposure)	Sum of biomarkers in CC was 5.4 times higher than HTP users Decreased VOCs and slightly increased TSNAs, 3HPMA, and 3HMPMA after switching from CC to HTP HTPs smoking for 2 weeks significantly reduced biomarker residues	Reduction in urinary VOC biomarkers after switching to HTPs indicate lower exposure to combustion-related toxicants. However, persistent TSNA exposure suggests continued exposure to tobacco-specific carcinogens, emphasizing that HTPs are not risk-free.
(69)	*n* = 60 (age: 28.1–36.1 years) CC, EC, HTP, oral tobacco and nicotine replacement therapy users, and non-smokers	Human bladder carcinogens o-Tol, 2-AN and 4-ABP, and 3-ABP were tested in urine sample (Biomarkers of Exposure)	Use of EC, HTP, nicotine gum and oral tobacco was not associated with significant exposure to o-Tol, 2-AN, 4-ABP and 3-ABP CC users had significantly elevated all biomarkers than non-smokers or non-CC users	Lower urinary aromatic amine biomarkers in HTP and EC users compared with CC smokers suggest substantially reduced exposure to bladder carcinogens associated with cigarette combustion.
(53)	*n* = 89 (age: 27.5 ± 8.4 years) CC smokers either continued or switched to HTP, or stopped smoking	HPHCs and pyrene were tested in urine and breath (Biomarkers of Exposure)	Except for NNAL and N-nitrosonornicotine, significant reductions in most biomarkers after 5 days of switching to HTP Except for NNAL and N-nitrosonornicotine, magnitude of reduction in other HPHCs in HTP group was similar to those who stopped smoking Relative to smoking continuation, switching to HTPs reduces risk	Rapid reductions in most HPHC biomarkers following switching to HTPs demonstrate decreased exposure to combustion-derived toxicants, although persistent NNAL and N-nitrosonornicotine indicate ongoing exposure to tobacco-specific nitrosamines.
(70)	*n* = 10 (age: 52.8 ± 9.8 years) exposed to 32-puffs of cigarette smoke, HTP or EC aerosols	Squalene, Squalene monohydroperoxide and malondialdehyde were tested in sebum samples (Biomarkers of Potential Harm)	Squalene monohydroperoxide, its ratio with squalene, and malondialdehyde levels were increased after exposure to cigarette smoke than HTP and EC Cigarette smoke causes a higher level of skin discolouration, and oxidation of sebum lipid and skin barrier lipids HTP and EC use has comparatively better cosmetic benefits over CC	Lower levels of oxidative damage biomarkers in sebum suggest reduced skin oxidative stress following HTP exposure compared with cigarette smoke; however, these findings primarily reflect cosmetic and localized biological effects rather than systemic health outcomes.
(71)	*n* = 568 (age: 45.4 ± 9.3 years) HTP and CC users, and non-smokers	Biomarker of nicotine exposure, lipid metabolism, vascular endothelial function, inflammation, platelet activation, oxidative stress, and HPHCs of smoking exposure were tested in urine sample (Biomarkers of Potential Harm)	11-DHTXB2 and 8-epi-PGF2α was similar in vaper users and non-smokers; 2,3-d-TXB2 was higher in vaper users than non-smokers Tobacco vaper users were less exposed to nicotine, and have significantly lower levels of triglyceride, sICAM-1, WBC count, 11-DHTXB2, 2,3-d-TXB2, 8-epi-PGF2α than CC smokers	Improvements in biomarkers of oxidative stress, inflammation, endothelial dysfunction, and platelet activation suggest a potentially more favourable cardiovascular risk profile among HTP users than CC smokers, although long-term clinical benefits remain uncertain.
(72)	*n* = 1,586 (age: 19–65 years) CC, EC and HTP cigarette users, and non-smokers	Urine nicotine, cotinine, OH-cotinine, NNAL, CYMA and CEMA were tested (Biomarkers of Exposure)	HTP cigarette users had similar nicotine, cotinine, OH-cotinine and CYMA levels as CC users EC users had lower biomarkers than HTP cigarette and CC users	Similar nicotine and exposure biomarkers between HTP and CC users indicate that HTPs can deliver nicotine at levels comparable to cigarettes, while reductions observed with ECs.
(52)	*n* = 285 (age: 37–40 years) CC smokers either continued, switched to HTP, or stopped, and non-smokers	Oxidative stress-, cardiovascular diseases-, respiratory diseases- and cancer-related biomarker of exposure and of potential harm were tested in urine and blood samples (Biomarkers of Exposure and Potential Harm)	NNAL reduced by 50% 30 days after switching to HTP and 80% after smoking cessation 8-epi-PGF2α reduced by 39% 180 days after switching to HTP and 17% after smoking cessation Significantly reduced 1-OHP, 2-AN, 4-ABP, 3HPMA, 3HMPMA, CEMA, eCO, HEMA, NNAL, MHBMA, o-Tol and S-PMA after 90 days of switching to HTP than who continued CC Deleterious health effects of CC may be reduced by complete switching to exclusive HTP use	Sustained reductions in biomarkers of exposure (including CEMA (a biomarker of acrylonitrile exposure), NNAL, and VOC metabolites) and biomarkers of potential harm following complete switching to HTPs indicate reduced exposure to several harmful combustion-related toxicants.

1-OHP, 1-hydroxypyrene; 2-AN, 2-aminonaphthalene; 2,3-d-TXB2: 2,3-dinor thromboxane B2; 3HMPMA, 3-hydroxy-1-methylpropylmercapturic acid; 3HPMA, 3-hydroxypropylmercapturic acid; 8-epi-PGF2α, 8-epi-prostaglandin F2α; 11-DHTXB2, 11-dehydrothromboxane B2; 3-ABP, 3-aminobiphenyl; 4-ABP, 4-aminobiphenyl; CC, Combustible cigarette; CEMA, 2-cyanoethylmercapturic acid; CYMA, N-acetyl-S-(2-cyanoehtyl)-L-cysteine; EC, Electronic cigarette; eCO, Exhaled carbon monoxide; HEMA, 2-hydroxyethylmercapturic acid; HPHC, Harmful and potentially harmful constituent; HTP, Heated tobacco product; MHBMA, Monohydroxybutenyl-mercapturic acid; NNAL, 4-(methylnitrosamino)-1-(3-pyridyl)-1-butanol; OH-cotinine, Trans-3′-hydroxycotinine; o-Tol, Ortho-toluidine; sICAM-1, Soluble intercellular adhesion molecule-1; S-PMA, S-phenylmercapturic acid; TSNA, Tobacco-specific nitrosamine; VOC, Volatile organic compound; WBC, White blood cells.

Overall, the studies summarized in Table 2 consistently demonstrate lower levels of several biomarkers of exposure among individuals who switch from CCs to HTPs, suggesting reduced exposure to a range of harmful and potentially harmful constituents. Improvements have also been reported for selected biomarkers of potential harm, including markers of oxidative stress, inflammation, and vascular dysfunction consistent with the proposed harm reduction profile of HTPs. Some studies indicate that HTP users continue to exhibit elevated nicotine exposure and biomarkers of DNA damage compared with non-smokers, highlighting that HTPs are not risk-free. Biomarkers are also surrogate indicators and do not necessarily predict long-term clinical endpoints, and the evidence is further limited by relatively small sample sizes and short follow-up periods in several studies. The evidence is also limited by relatively small sample sizes in several studies, short follow-up periods. The available biomarker data support reduced exposure to several toxicants relative to continued CC smoking under some switching conditions. Biomarkers are surrogate measures, however, these findings should not be interpreted as demonstrating reduced clinical disease or mortality. Independent longitudinal studies are required to determine whether exposure reductions translate into lower disease incidence or mortality. Moreover, some of these influential studies were also industry-affiliated. Gale et al. (52) was funded by British American Tobacco and authored largely by company employees or consultants; Yuki et al. (53) was sponsored by Japan Tobacco Inc. and authored by company researchers; and most authors of Li et al. (54) were affiliated with the Shanghai New Tobacco Product Research Institute Co., Ltd. Though these links do not automatically invalidate the findings, but they do warrant transparent reporting and independent replication.

### 2.6 Public health regulatory frameworks for harm reduction

Global estimates for young (aged 15–24 years) tobacco smokers were 155 million in 2019, comprising 20.1% males and 4.95% females (55). Surveys from 53 African countries recorded 14.3% prevalence of any tobacco use among adolescents; prevalence for CC and smokeless tobacco was 6.4% each, for other smoked tobacco was 6.7%, and for dual use of CC and smokeless tobacco was 3.0% (56). A school-based youth risk behaviour survey, conducted in US, reported a peak use in 2019 at over 5 million users, declined through 2021, and has since stabilized (74). In 2024, 2.25 million middle and high school students reported current use (57–60). As a majority of smokers start smoking before the age of 20 years, countries can put efforts in preventing next generation from smoking by substantial implementation and enforcement of tobacco control policies (55).

Health could be protected by government-implemented comprehensive tobacco control rules that lower supply and demand for all tobacco/ nicotine products (61). The World Health Organization (WHO) introduced a holistic policy framework MPOWER (M: monitor tobacco use, P: protect people from tobacco smoke, O: offer help to quit, W: warn about the dangers of tobacco, E: enforce bans on tobacco advertising, promotion and sponsorship, R: raise tobacco tax) to help countries implement and manage tobacco control. A quantitative analysis of the MPOWER reflected a considerably successful implementation and enforcement of the tool for tobacco control by many countries of South-East Asian region. However, data revealed minimal adherence to smoke-free regulations, particularly regarding the advertisements (62). Increased public awareness about the adverse effects of HTPs is essential (36). Policymakers should assess tolerance for nicotine threshold and lifetime cancer risk to make a total tobacco-free space (28).

While HTPs may offer harm reduction benefits for adult smokers, it is important that marketing and availability of alternative tobacco and nicotine products are appropriately regulated, particularly to protect adolescents and young adults from tobacco initiation (13). Therefore, regulatory frameworks should balance the potential harm reduction benefits for current smokers with robust measures aimed at preventing uptake among youth and other vulnerable populations. Evidence suggests that the perceived reduced-harm profile of HTPs, alongside product innovation and marketing strategies, may attract use among individuals who have not previously used tobacco. While this consideration is relevant to public health policy, it should be balanced against the established harm reduction potential of HTPs for current adult smokers who would otherwise continue to use combustible cigarettes. Careful considerations are required as people are negligent of the opportunity for harm reduction. Removal of harmful products from the market may not eliminate the demand, rather can push the users to seek illicit channels (8). Regulatory measures could be implemented to restrict the marketing, widespread sale and affordability of HTPs, especially in adolescents (36).

## 3 Conclusion

Heated tobacco products (HTPs) represent a distinct category of non-combustible tobacco products that may be used by adults who otherwise continue smoking conventional cigarettes (CCs). Complete cessation remains the preferred outcome However, even though relative to continued CC smoking, complete switching to HTPs may reduce exposure to several combustion-related toxicants, but current evidence does not demonstrate reduced cancer, cardiovascular disease, chronic obstructive pulmonary disease, or mortality, and it does not establish HTPs as effective cessation interventions. Any net population-health effect would depend on sustained switching, relapse, dual or poly use, youth and non-smoker initiation, and displacement of evidence-based cessation. These products maybe of lower exposure but are not entirely risk-free as an alternative to CCs. Hence, these findings should be interpreted with caution, as the existing evidence base is largely derived from short-term experimental and observational studies, and much of the available literature originates from a limited number of countries. Consequently, the long-term health effects of HTP use and the generalizability of current findings across diverse populations and regulatory settings remain uncertain. Long-term epidemiological study from different countries, reporting sex-stratified outcomes, comparing HTPs with CCs is essential for quantifying the relative health risks and guide evidence-based public health policy. Future research should also address critical knowledge gaps related to nicotine pharmacology, dependence, withdrawal, relapse, comparative abuse liability, dual- and poly-use patterns, as well as youth uptake, including the influence of flavored products, social media, cannabis co-use, and the effects of nicotine exposure on the developing adolescent brain. Integrating public health strategies related to HTPs within national development priorities can help address tobacco-related disease burdens and reduce healthcare costs. Policymaking should have a balance between harm reduction for individuals and population, while restricting the new users.
